# Mechanical and thermal thresholds before and after application of a conditioning stimulus in healthy Göttingen Minipigs

**DOI:** 10.1371/journal.pone.0309604

**Published:** 2024-08-29

**Authors:** Mariafrancesca Petrucci, Claudia Spadavecchia, Robert Rieben, Daniela Casoni

**Affiliations:** 1 Faculty of Medicine, Experimental Surgery Facility (ESF), Experimental Animal Center (EAC), University of Bern, Bern, Switzerland; 2 Faculty of Medicine, Department for BioMedical Research, University of Bern, Bern, Switzerland; 3 Graduate School for Cellular and BioMedical Science, University of Bern, Bern, Switzerland; 4 Vetsuisse Faculty, Department of Clinical Veterinary Medicine, Anaesthesiology and Pain Therapy Section, University of Bern, Bern, Switzerland; University of Minho, PORTUGAL

## Abstract

Minipigs are widely used in biomedical research for translational studies. However, information about pain elicited by experimental procedures is lacking. Non-invasive methods as quantitative sensory testing and conditioned pain modulation are particularly attractive. Our overarching aim was to explore and refine these methods for assessing post-operative pain in minipigs after myocardial infarction. As first step, we aimed at defining mechanical and thermal thresholds in healthy adults Göttingen Minipigs, evaluating their reliability, and testing their modifications after the application of a conditioning stimulus. Thresholds were assessed at different body sites before and after a painful conditioning stimulus (CS) (cuffed tourniquet) and sham CS (uncuffed tourniquet) in eleven animals. Thresholds’ reliability was assessed using interclass correlation coefficient (ICC). The effect of the CS was assessed calculating absolute change, percentage change of the thresholds and standard error of measurement. Baseline mechanical thresholds (Newton) were: left hindlimb 81 [73; 81]; left forearm 81 [72.1; 81]; right forearm 81 [76; 81]; left chest 80.5 [68; 81]; right chest 81 [76.5; 81]; left neck 81 [70.3; 81]; right neck 74.8 [62.3; 80.5]. Reliability of mechanical thresholds was good at right chest (ICC = 0.835) and moderate at left chest (ICC = 0.591), left hindlimb (ICC = 0.606) and left neck (ICC = 0.518). Thermal thresholds showed poor reliability in all the tested sites. A modulatory effect was present at right chest, but it was seen when both a painful CS and a sham CS was applied. Minipigs tendentially showed a pro-nociceptive profile (i.e. conditioning pain facilitation). The measured thresholds are a reference for future trials in this species. Mechanical thresholds showed to be more reliable and, therefore, more useful, than thermal ones. The pain facilitation might be explained by the phenomenon of stress induced hyperalgesia, but this finding needs to be further investigated with a stricter paradigm.

## Introduction

Pigs and minipigs are widely used in biomedical research for translational studies due to multiple anatomical and physiological analogies with humans [1–3]. Although similarities in the pain pathophysiology might be also expected, little information about pain detection and quantification in pigs is available [2] and no information in minipigs is present.

Quantitative sensory testing (QST) are non-invasive methods to evaluate somatosensory sensitivity of the pain pathway [4]. They provide insight into the functioning of the Aδ and C fibres, which are responsible for transmitting the nociceptive signal from the periphery to the central nervous system [4]. These methods have been widely used both in humans and in veterinary medicine to quantify pain thresholds [5–13] and to evaluate their changes following the administration of analgesic treatments [14–16]. By investigating the reliability of QST thresholds, specifically their consistency across time, patients and observers, it is possible to enhance validity of the research findings and to establish reliable baseline values, which could be used as reference in future studies [17–19]. To the authors’ knowledge, no information regarding either QST thresholds in healthy minipigs and their reliability is currently available in literature.

Once a nociceptive stimulus reaches the central nervous system, a modulation [20] via the activation of the descending pain pathway occurs [21]. The modulation can be evaluated through the so-called conditioned pain modulation (CPM) paradigm [21]: simultaneously or immediately after a nociceptive stimulus (i.e. test stimulus), a second nociceptive stimulus (i.e. conditioning stimulus) is applied in a different and remote area of the body, and a modification of the perception of the test stimulus, therefore its threshold, is expected [22]. Both an inhibitory (reduced sensation) or facilitatory (increased sensation) effect can be elicited [21]. Several studies investigating CPM in humans have been carried out [23–27]. In veterinary medicine, only a few studies in dogs [28,29], cats [30,31], calves [5], mice [32–35] and rats [36] have been published. No information is currently present for minipigs.

Our overarching aim was to explore and refine these methods for a future assessment of post-operative pain in minipigs undergoing myocardial infarction, a possible source of cardiac visceral pain. As first step, with this study we aimed at: 1) defining mechanical and thermal nociceptive thresholds in healthy adult Göttingen Minipigs; 2) evaluating their reliability in different body areas; 3) investigating the modulatory effect of a conditioning stimulus.

We hypothesised that: 1) mechanical and thermal thresholds could be defined; 2) the most reliable sites for an appropriate QST assessment would be identified; 3) the conditioning stimulus would have affected mechanical and thermal thresholds and both inhibition or facilitation could have occurred in individual animals.

## Materials and methods

### 1. Study design and ethical approval

The present study was designed as prospective, cross-control, randomised experimental study. It was reviewed and approved by the Committee for Animal Experiments of the Canton of Bern, Switzerland (national permission number: 33492). For reporting all the experimental procedures, the ARRIVE guidelines (Animals in Research: Reporting of In Vivo Experiments) have been strictly followed [37].

### 2. Animals

Eleven healthy Göttingen Minipigs (five castrated males and six females), aged 15 ± 2 months and weighed 25 ± 3 kg were enrolled.

The animals were purchased from their official breeder, Ellegaard Göttingen Minipigs (Dalmose, Denmark) and transported to Switzerland. The animals were hosted in group of six to ten in an 18 m^2^ pen with a 50 m^2^ outdoor area located on a farm. Details related to the pen and its surroundings are reported in the supplementary materials (S1 Fig). Enrichment was provided with straw and toys (hanging ropes and balls). Water was available ad libitum, whereas food (800 grams/minipig/day) (Minipigs Maintenance Standard, Kliba Nafag, Switzerland) was provided twice per day. At least one week of acclimatisation was allowed before starting the trial, during which the animals were accustomed to the presence of researchers and caretakers, and to manipulations and environment. In particular, experimenters and caretakers spent one to two hours per day with the animals. During this time, the animals learnt to approach humans without fear and were trained with rewarding (apple juice or apple mousse) to touch, clinical exams, and to comfortably moving onto a scale for body weight measurement. Moreover, all minipigs were placed in a sling (Lomir Biomedical Inc., Canada) and transported at least once before the beginning of the trial into the experimental room where they were rewarded for improving their acceptance of the procedure. Health checks, including physiological and behavioural evaluations, were conducted once per day between 9:00 AM and 6:00 PM. Body weight was measured once per week to promptly detect any potential weight loss.

### 3. Course of the experiment

**Fig 1:**
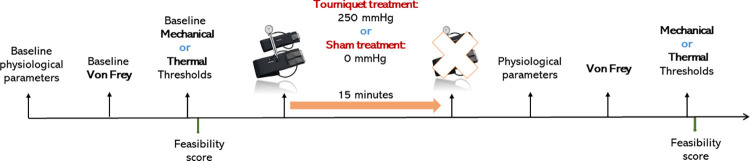
Timeline of the experiment.

A timeline of the trial, showing the course of procedures performed in each single session (one day session) is showed in Fig 1.

### 3.1 Quantitative sensory testing (QST) thresholds

Sessions were always performed with animals recently fed (at least one hour before), for improving compliance toward manipulations. The minipigs were placed in the sling and conducted into the experimental room, adjacent to their pen, where the trials were carried out. Each experimental day, minimum one and maximum six animals were tested at different times. During each session, minipigs were positioned on the sling and special food (apple juice or apple mousse) was offered as rewarding at the beginning and at the end of the procedure. At least one day of wash out between sessions was guaranteed.

All the minipigs underwent six experimental sessions in six different days:

■ Mechanical—tourniquet (MT): mechanical thresholds were assessed before (baseline) and after the application of an ischemic conditioning stimulus (CS). This session was repeated twice in two different days (MT1 and MT2).
■ Mechanical—sham (MS): mechanical thresholds were assessed before (baseline) and after the application of a sham ischemic CS. This session was repeated twice in two different days (MS1 and MS2).
■ Thermal—tourniquet (TT): thermal thresholds were assessed before (baseline) and after the application of an ischemic CS.
■ Thermal—sham (TS): thermal thresholds were assessed before (baseline) and after the application of a sham ischemic CS.

During all the session, the presence of allodynia was also assessed (see below).

The animals were randomly assigned to one session at a time (www.randomization.com).

**Fig 2:**
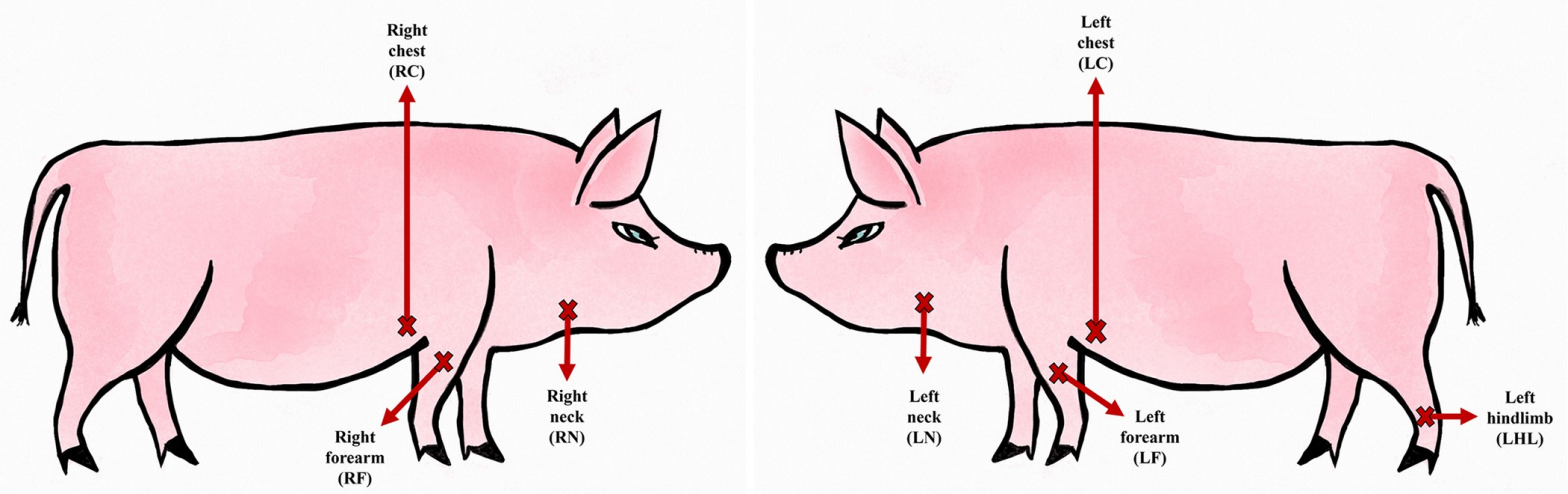
Representation of the tested sites on the right and left side of the minipigs.

The QST thresholds were assessed at different body sites (Fig 2), as follows:

- Left hindlimb (LHL): midway over the metatarsus, dorsally; left hindlimb only
- Right and left forearm (RF, LF): midway over the extensor carpi radialis muscle
- Right and left chest (RC, LC): over the lower third of the chest, around five centimetres behind the olecranon.
- Right and left neck (RN, LN): the intersection of two lines was considered: a first line perpendicular to the ground was drawn two centimetres behind the ear base; a second line parallel to the ground was drawn at the level of the shoulder.

All sites were assessed in each session in a random order.

Thresholds to both mechanical and thermal stimulations were defined at the value measured when the animals showed:

- withdrawal of the tested limb/area, escape reactions (rapid and voluntary body movement away from the source of stimulation).
- vocalisation and watching in the direction of the tested area.
- muscle twitch at the level of the tested area with head turned in that direction.

The area to test was pinpointed and marked with a dermographic pen to ensure consistent testing of the same area across different sessions.

To assess mechanical thresholds, a manual hand-held mechanical algometer (FPN 100–Algometer, Wagner Instruments, Greenwich, Connecticut, USA) with a contact area 1 cm^2^ (rubber tip) was used. After positioning of the tip on the body site to test, force was applied perpendicular to the skin, aiming at a constant rate increase of 2 Newton/second. Cut-off stimulation was set at 80 Newton (N) (800 Kilopascal). Mechanical thresholds were recorded twice per session at each site, and the sites were tested with the same order. If the difference between two recorded thresholds was more than 20% of the lowest threshold, a third assessment was carried out, and the two nearest values were considered for statistical analysis. A standard interval of 30 seconds was applied among assessments, to guarantee lack of residual effects from previous measurement and still maintain the time of each session reasonably short.

To assess thermal thresholds, a self-constructed thermal stimulator equipped with a probe having a contact area of 1 cm and delivering heat stimulus was used. After positioning the probe on the tested site, the stimulus was initiated. The rate of heat stimulus increase was set at 0.6–0.8°C per second using a manual gearwheel. Once the probe was placed on the skin, the device was activated, delivering increasing heat stimuli until the animal exhibited a reaction. Stimulation cut-off was 55°C (device uppermost limit and safety cut-off to prevent skin damage). Differently from mechanical thresholds, thermal thresholds were recorded only once per site to avoid risk of burning.

To assess allodynia, a set of Von Frey monofilaments (BioSeb Lab, France) (from 1 to 300 g/cm^2^) were used. The monofilaments were applied on each site from the thinnest to the thickest, selecting 11 sizes. They were positioned on the skin at a 90° angle and pressed until warped. Then, they were kept in position for 1.5 seconds and finally retracted. As for the mechanical thresholds, allodynia was tested twice per site.

For the Von Frey monofilaments test, a positive response was defined when the animal reacted twice to the same filament, or when it reacted once, but at two subsequent monofilaments. In the latter case, the highest value was considered (e.g., one response at 180 g/cm^2^ and one response at 300 g/cm^2^ = threshold at 300 g/cm^2^).

### 3.2 Assessment of thresholds after conditioning stimulus

Immediately after baseline thresholds recording, a conditioning stimulus was applied. As CS a pneumatic tourniquet was used (cuff width: 28 cm (11.02 in) x 6 cm (2.36 in)), Komprimeter Pneumatic Tourniquet, Rudolf Riester GmbH). The tourniquet was positioned on the metatarsal area on the left hindlimb and kept in position for 15 minutes. Depending on the treatment (sham versus tourniquet), cuff was either cuffed to 250 mmHg (MT1, MT2, TT) or kept uncuffed (MS1, MS2, TS). After 15 minutes, tourniquet was deflated and allodynia first and then the mechanical or thermal thresholds were assessed.

To increase repeatability and reduce detection bias, the same investigator (MP) assessed all the thresholds, while another investigator was responsible of filling out the experimental sheet.

### 3.3 Other recorded variables

A clinical examination was performed to obtain baseline heart rate (HR), respiratory rate (RR) and temperature. The variables were recorded before thresholds assessment (baseline) and immediately after CS removal.

A “feasibility score” was recorded at the end of each session (MT1, MT2, MS1, MS2, TT and TS). A purpose-made score was used (Table 1) and a score (ranging from 0 to 3) was assigned for assessing animals’ reaction when thresholds were recorded both at baseline and after the removal of CS. Overall, this served to assess animals’ compliance toward the manipulations. If an animal had a score of 3 after baseline thresholds assessment (indicating the inability to distinguish clear response to test stimuli from excitation) the session was stopped and postponed.

**Table 1:** Feasibility score system.

Feasibility scores	
**0**	No excitation reactions to manipulation (i.e. clear reaction to stimulus in ≥ 90% cases)
**1**	Mild excitation reactions to manipulation (i.e. clear reaction to stimulus in ≥ 75% cases)
**2**	Moderate excitation reactions to manipulation (i.e. clear reaction to stimulus in ≥ 50% cases)
**3**	Intolerant to any manipulation (i.e. clear reaction to stimulus in less than 50% cases)

At the beginning each session, the animals were rewarded (with apple juice or apple mousse) and at end of each session, they were rewarded again before being brought back into their pen.

Start of the session, end of the session, time of CS positioning and removal were recorded for each session and animal on the experimental sheet.

### 4. Statistical analysis

Statistical analysis was performed using Sigma Stat v4.0 (Inpixon HQ, USA) and R Studio Statistical Software (version 4.3.2; R Foundation for Statistical Computing, Austria). Normality was assessed using the Shapiro Wilk test, and results are presented as mean ± standard deviation (SD) (normally distributed data) or median and interquartile range (IQR) [25^th^; 75^th^] (not-normally distributed data).

#### 4.1 Quantitative sensory testing (QST) thresholds

When the cut-off was reached before a reaction of the animals, mechanical and thermal thresholds were recorded as 81 N and 56°C respectively.

For mechanical thresholds, the arithmetic mean of the two recorded values, for each tested site, was calculated and used for further analysis.

Median and IQR of the baseline mechanical and thermal thresholds (independently from the treatment) per each site were calculated.

#### 4.2 Reliability

Reliability of the baseline thresholds was assessed using the interclass correlation (ICC 3,1, two-way mixed effect, single rater, single measurement, consistency), and the 95% confidence intervals was determined. Interclass correlation was calculated between T and S treatments (MT1+ MT2 versus MS1+ MS2 (22 values), and TT versus TS (11 values)). An ICC < 0.5 was considered as poor, between 0.5 and 0.75 as moderate, values between 0.75 and 0.9 as good, and values > 0.90 as excellent [38].

#### 4.3 Assessment of thresholds after conditioning stimulus

For assessing the modulation of the conditioning stimulus, statistical methods recommended for analysing CPM in human medicine were applied [25,39–41]. Moreover, as the modulation effect should be assessed in remote sites from the CS, the LHL was excluded from this analysis. To evaluate the effect of CS on recorded mechanical thresholds, two datasets including the thresholds recorded in treatments MT1 and MT2 (named group T = 22 measurements), and in treatments MS1 and MS2 (named group S = 22 measurements) were generated. To assess the extent of thresholds’ variation, the absolute change, and the percentage (%) change were calculated for group T and group S, as follows:

Mechanical and Von Frey monofilaments thresholds:

Absolutechange=meanthresholdspostCS−meanthresholdspreCS

$$\%\;change=\frac{absolute\;change}{mean\;thresholds\;pre\;CS}*100$$

Thermal thresholds:

Absolutechange=rawthresholdpostCS−rawthresholdpreCS

$$\%\;change=\frac{absolute\;change}{raw\;threshold\;pre\;CS}*100$$

To evaluate if habituation occurred, differences in absolute change between MT1 and MT2, and MS1 and MS2 were assessed for each site using the Mann-Whitney Signed Rank test.

To establish the range of meaningful modulation, the Standard Error of Measurement (SEM) was calculated using the baseline mechanical and thermal thresholds [39–41], and one value per treatment was calculated (group T: MT1+ MT2, 22 measures; group S: MS1+ MS2, 22 measures; TT, 11 measures; TS, 11 measures).

The following formula was used to calculate SEM:

$$SEM=SD\;of\;baseline\;thresholds*\;\sqrt{1}-ICC\;baseline\;threholds$$

SD = standard deviation.

In agreement with the human guidelines regarding CPM [37,38], a meaningful modulation was defined at reliable sites but LHL as: a) inhibitory: increase in thresholds > 2 * SEM; b) facilitatory: decrease in thresholds > 2* SEM; c) no effect: variation in thresholds < 2 * SEM The mean absolute change of those animals showing facilitation, inhibition and no modulatory effect was calculated.

Number of minipigs exhibiting a modulatory effect or no modulatory effect was compared between treatments (group T (MT1+MT2) versus group S (MS1+MS2); TT versus TS) for each site using the Fisher’s exact test. Only sites resulted to have moderate to good reliability were considered for this comparison.

#### 4.4 Other parameters

Values of HR, RR, body temperature and feasibility scores before versus after CS were compared using the Wilcoxon signed rank test, and between treatments, before and after CS, using the Mann Whitney signed rank test.

Duration of the experimental sessions was calculated as:

Endofthesession(time)−startofthesession(time)

Duration of thresholds assessment after CS removal (which corresponds to the time needed to assess modulation) was calculated as:

Endofthesession(time)−Removaloftourniquet(time)

Comparisons among durations were performed using the Kruskal-Wallis One Way ANOVA on Ranks for MT1, MT2, MS1 and MS2, and the Mann-Whitney Rank Sum Test for TT and TS.

Statistically significant difference was set at p < 0.05.

## Results

All the enrolled minipigs were included in the final data analysis. One baseline value in MT1 at LHL and one after removal of CS in TT at RF were not recorded (investigator in charge of recording data did not report them on the experimental sheet). Data collection started in December 2021 and terminated in February 2023.

### 1. Quantitative sensory testing (QST) thresholds

Raw mechanical and thermal thresholds recorded in each tested site and for each session are reported in Tables 2 and 3, and divided per gender in S1–S4 Tables.

**Table 2:** *Raw mechanical thresholds*, *before and after the application of the conditioning stimulus (CS)* Results (in Newton) are presented as median and interquartile range [25^th^; 75^th^].

SITE	Time point	MT1 (n = 11)	MT2 (n = 11)	MS1 (n = 11)	MS2 (n = 11)
**LHL**	Before CS*	**81** *[80*.*9; 81]*	**80** *[67*.*5; 81]*	**81** *[78*.*5; 81]*	**73** *[59*.*5; 81]*
	After CS	**81** *[65*.*5; 81]*	**66** *[50; 69]*	**66,5** *[57*.*5; 77*,*5]*	**71** *[54; 78*.*5]*
**LF**	Before CS	**81** *[72*.*5; 81]*	**79** *[67*.*5; 81]*	**81** *[75; 81]*	**79.5** *[61*.*5; 81]*
	After CS	**81** *[65*.*5; 81]*	**80,5** *[62; 81]*	**81** *[75*.*5; 81]*	**81** *[76*.*5; 81]*
**RF**	Before CS	**81** *[81; 81]*	**78.5** *[74; 81]*	**81** *[75*.*5; 81]*	**81** *[77*.*5; 81]*
	After CS	**71** *[63; 80]*	**70.5** *[52; 81]*	**79.5** *[73*.*5; 81]*	**79.5** *[51; 81]*
**LC**	Before CS	**72.5** *[67; 81]*	**80** *[55*.*5; 81]*	**81** *[72*.*5; 81]*	**80.5** *[65*.*5; 81]*
	After CS	**79** *[72*.*5; 81]*	**65.5** *[45; 77]*	**68** *[48; 78*.*5]*	**66** *[56*.*5; 79*.*5]*
**RC**	Before CS	**81** *[78; 81]*	**81** *[73*.*5; 81]*	**81** *[74; 81]*	**81** *[76*.*5; 81]*
	After CS	**77.5** *[43; 79*.*5]*	**66** *[48; 71]*	**76** *[63; 79*.*5]*	**72** *[58*.*5; 79]*
**LN**	Before CS	**71.5** *[67; 81]*	**77.5** *[51; 81]*	**81** *[79*.*6; 81]*	**81** *[69*.*5; 81]*
	After CS	**79.5** *[74*.*5; 81]*	**75.5** *[60; 81]*	**78.2** *[68*.*4; 81]*	**78.5** *[68; 81]*
**RN**	Before CS	**75.5** *[62; 81]*	**70** *[44*.*5; 76]*	**76.5** *[60*.*5; 81]*	**74.5** *[69*.*5; 81]*
	After CS	**73.5** *[65*.*5; 80*.*5]*	**63.5** *[52; 75]*	**75** *[64*.*5; 81]*	**80** *[57; 81]*

Mechanical thresholds are reported in all the tested sites (LHL: Left hindlimb, LF: Left forearm, RF: Right forearm, LC: Left chest, RC: Right chest, LN: Left neck, RN: Right neck) both before and after the application of the CS in all the sessions (MT1: Mechanical tourniquet 1, MT2: Mechanical tourniquet 2, MS1: Mechanical sham 1, MS2: Mechanical sham 2). * One missing value (n = 10).

**Table 3:** Raw values of thermal thresholds, before and after the application of the conditioning stimulus (CS).

SITE	Time point	TT (n = 11)	TS (n = 11)
**LHL**	Before CS	**45.4** *[44*.*1; 47]*	**48.3** *[44*.*3; 51]*
	After CS	**45.1** *[44*.*4; 48*.*2]*	**47.4** *[44*.*2; 52*.*9]*
**LF**	Before CS	**49.6** *[45*.*4; 55]*	**48.9** *[44*.*6; 50*.*8]*
	After CS	**47.4** *[44*.*8; 51*.*2]*	**47.6** *[44*.*2; 53*.*8]*
**RF**	Before CS	**50.4** *[45*.*7; 56]*	**52.1** *[44*.*7; 55*.*1]*
	After CS	**50.5** *[43*.6; 56]*	**52.7** *[46*.*6; 56]*
**LC**	Before CS	**43.3** *[41*.*9; 49]*	**44.6** *[43*.*1; 45*.*1]*
	After CS	**44.3** *[42*.*9; 45*.*5]*	**43.5** *[42*.*4; 43*.*9]*
**RC**	Before CS	**45.3** *[43*.*1; 48]*	**43.7** *[42*.*9; 48*.*1]*
	After CS	**44.3** *[43; 48*.*2]*	**43.8** *[43*.*4; 46*.*2]*
**LN**	Before CS	**44.6** *[43*.*5; 51*.*3]*	**45.7** *[44*.*3; 48*.*3]*
	After CS	**45.7** *[42*.*7; 50*.*2]*	**47** *[44*.*6; 47*.*9]*
**RN**	Before CS	**47.8** *[45*.*9; 51]*	**48.7** *[44*.*9; 55]*
	After CS	**48.5** *[45*.*5; 55]*	**48.5** *[44*.*7; 55]*

Results (degrees Celsius) are presented as median and interquartile range [25^th^; 75^th^]. Thermal thresholds are reported in all the tested sites (LHL: Left hindlimb, LF: Left forearm, RF: Right forearm, LC: Left chest, RC: Right chest, LN: Left neck, RN: Right neck) both before and after the application of the CS in all the sessions (TT: Thermal tourniquet; TS: Thermal sham). * One missing value (n = 10).

Mean and SD of the rate of probe temperature rise was 0.65 ± 0.13°C/second.

Median and IQR of the baseline mechanical and thermal thresholds, independently from the treatment, in the whole sample and in the two different genders (females and males) are reported in Tables 4 and 5.

**Table 4:** Raw baseline mechanical thresholds values in both sex and in the two different genders (females and males) in all treatments.

Site	BOTH SEXES (n = 11)		FEMALES (n = 6)		MALES (n = 5)	
	Median (n = 44)	Interquartile range [25^th;^ 75^th]^	Median (n = 24)	Interquartile range [25^th;^ 75^th]^	Median (n = 20)	Interquartile range [25^th;^ 75^th]^
**LHL**	81	*[73, 81]*	81	*[72*.*7–81]*	81	*[78–81]*
**LF**	81	*[72*.*1; 81]*	81	*[70*.*9–81]*	81	*[73*.*1–81]*
**RF**	81	*[76*.*5; 81]*	81	*[77*.*8–81]*	81	*[75*.*5–81]*
**LC**	80.5	*[68; 81]*	80.5	*[66*.*1–81]*	80.3	*[68*.*3–81]*
**RC**	81	*[76*.*5; 81]*	81	*[74*.*9–81]*	81	*[76*.*9–81]*
**LN**	81	*[70*.*3; 81]*	81	*[69*.*6–81]*	81	*[71*.*1–81]*
**RN**	74.8	*[62*.*3; 80*.*5]*	75.3	*[69*.*5–81]*	73.8	*[60*.*9–77*.*5]*

Results (Newton) are presented as median and interquartile change [25^th^; 75^th^].

**Table 5:** Raw baseline thermal thresholds values in both sex and in the two different genders (females and males) in all treatments.

Site	BOTH SEXES (n = 11)		FEMALES (n = 6)		MALES (n = 5)	
	Median (n = 22)	Interquartile range [25^th;^ 75^th]^	Median (n = 12)	Interquartile range [25^th;^ 75^th]^	Median (n = 10)	Interquartile range [25^th;^ 75^th]^
**LHL**	45.9	*[44*.*2; 49*.*2]*	47.6	*[44*.*1–50]*	45.6	*[44–47*.*8]*
**LF**	49	*[45*.*4; 51*.*5]*	49.2	*[44*.*8–52]*	49	*[46*.*9–52]*
**RF**	51.3	*[45*.*6; 55*.*3]*	47.7	*[44*.*9–54*.*3]*	54.4	*[46*.*4–56]*
**LC**	44.8	*[42*.*6; 48]*	44.8	*[42*.*4–53*.*4]*	44.3	*[42*.*6–45*.*3]*
**RC**	45	*[43; 48]*	45.9	*[43*.*7–53*.*4]*	43.3	*[42*.*9–45*.*6]*
**LN**	45.5	*[44*.*1; 48*.*4]*	45.2	*[44*.*3–48*.*7]*	46.4	*[43*.*5–48*.*4]*
**RN**	47.9	*[45*.*4; 54*.*8]*	47.9	*[45–53*.*8]*	49	*[46*.*2–55]*

Results (degrees Celsius) are presented as median and interquartile change [25^th^; 75^th^].

Most of the animals did not respond to any Von Frey monofilaments (details reported in S5 Table). Thus, no further analysis was performed.

### 2. Reliability

For mechanical thresholds, the only site showing a good reliability was RC, while moderate reliability was found in LHL, LC and LN. The other sites, as well as all the sites for the thermal thresholds had a poor reliability. Interclass correlations (ICC 3,1) and 95% confidence intervals are reported in Tables 6 and 7 for mechanical and thermal thresholds, respectively.

**Table 6:** Interclass correlations (ICC) (3,1) and 95% confidence intervals of baseline mechanical thresholds.

Sites	Treatments	ICC (3,1)	95% confidence interval	Interpretation
**LHL**	MT1, MT2, MS1, MS2	0.606	0.3–0.86	**MODERATE**
**LF**	MT1, MT2, MS1, MS2	0.153	-0.085–0.122	POOR
**RF**	MT1, MT2, MS1, MS2	0.166	-0.077–0.553	POOR
**LC**	MT1, MT2, MS1, MS2	0.591	0.299–0.843	**MODERATE**
**RC**	MT1, MT2, MS1, MS2	0.835	0.651–0.946	**GOOD**
**LN**	MT1, MT2, MS1, MS2	0.518	0.218–0.806	**MODERATE**
**RN**	MT1, MT2, MS1, MS2	0.413	0.115–0.744	POOR

Results are reported for each tested site (LHL: Left hindlimb, LF: Left forearm, RF: Right forearm, LC: Left chest, RC: Right chest, LN: Left neck, RN: Right neck) Baseline values were recorded during session mechanical tourniquet 1 (MT1), mechanical tourniquet 2 (MT2), mechanical sham 1 (MS1) and mechanical sham 2 (MS2).

**Table 7:** Interclass correlations (ICC) (3,1) and 95% confidence intervals of baseline thermal thresholds.

Sites	Treatments	ICC (3,1)	95% confidence interval	Interpretation
**LHL**	TT and TS	-0.179	-0.687–0.711	POOR
**LF**	TT and TS	-0.214	-0.703–0.413	POOR
**RF**	TT and TS	-0.251	-0.722–0.38	POOR
**LC**	TT and TS	-0.145	-0.665–0.47	POOR
**RC**	TT and TS	0.00395	-0.537–0.579	POOR
**LN**	TT and TS	-0.0198	-0.589–0.563	POOR
**RN**	TT and TS	-0.625	-0.883–0.076	POOR

Results are reported for each tested site (LHL: Left hindlimb, LF: Left forearm, RF: Right forearm, LC: Left chest, RC: Right chest, LN: Left neck, RN: Right neck) Baseline values were recorded during sessions thermal tourniquet (TT) and thermal sham (TS).

### 3. Assessment of thresholds after conditioning stimulus

Only results related to sites with good and moderate reliability (LC, RC, LN, only for the mechanical thresholds) are shown below. The other results are reported in supplementary materials (S2-S5 Figs, S9 Table)

Absolute changes and % changes after CS, as well as results of SEM and 2* SEM are also reported in supplementary materials (S6–S8 Tables).

On a total sample of 22, a meaningful modulatory effect [25] was seen in:

- LC: 5 animals in group T (12%) and 8 in group S (36%)
- RC: 15 animals in group T (68%) and 16 in group S (55%)
- LN: 2 animals in group T (9%) and 2 in group S (9%)

**Fig 3:**
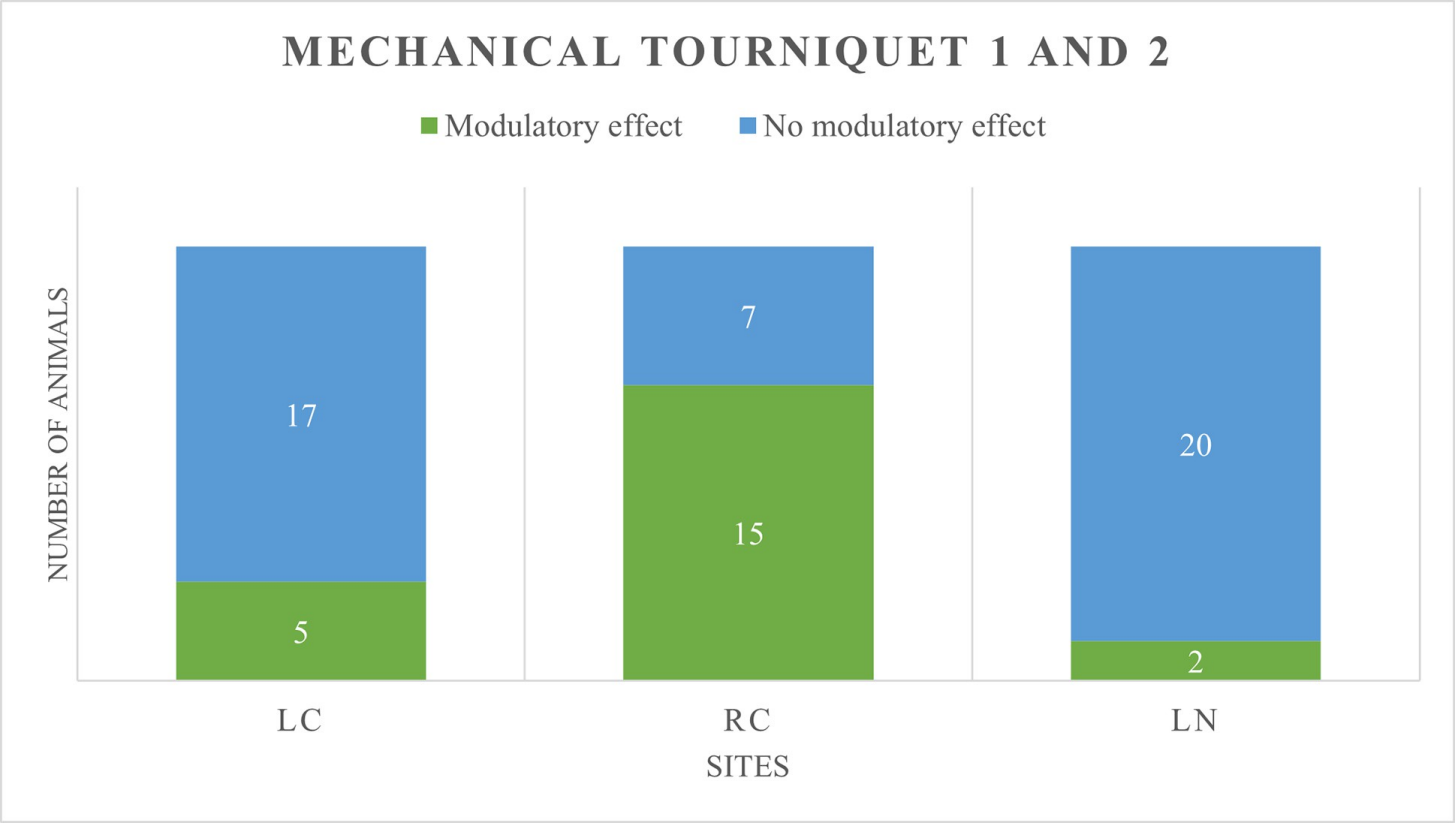
Number of minipigs exhibiting a modulatory effect (in green) or no modulatory effect (in blue) of the conditioning stimulus at treatment mechanical tourniquet 1 and 2, in the reliable tested sites. ***(***LC: Left chest, RC: Right chest, LN: Left neck).

**Fig 4:**
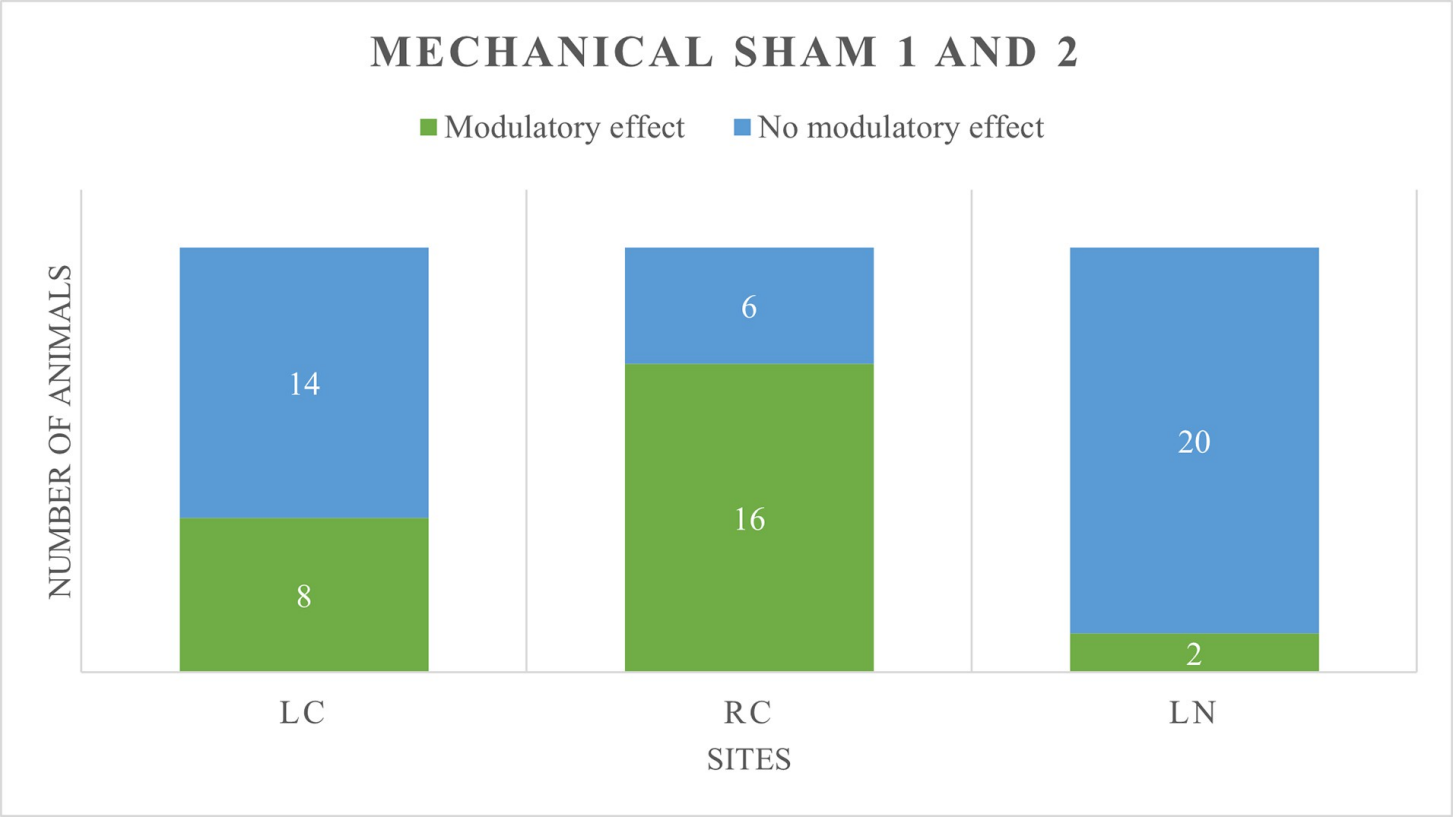
**Number of minipigs exhibiting a modulatory effect (in green) or no modulatory effect (in blue) at treatment mechanical sham 1 and 2, in the reliable tested sites** (LC: Left chest, RC: Right chest, LN: Left neck).

Results are shown in Figs 3 and 4.

Number of minipigs showing facilitation, inhibition, or no effect, as well as mean absolute change for each session is reported in Table 8.

**Table 8:** Number of minipigs showing facilitation, inhibition or no effect, and relative mean absolute change following the removal of the conditioning stimulus.

Site	Treatment	FACILITATION		INHIBITION		NO EFFECT	
		Number	Mean absolute change	Number	Mean absolute change	Number	Mean absolute change
**LC**	MT1, MT2	3	-32.7	2	28.8	17	-0.4
	MS1, MS2	8	-22.3	0	//	14	-1
**RC**	MT1, MT2	15	-20	0	//	7	-0.4
	MS1, MS2	12	-20.3	4	18.5	6	-1.3
**LN**	MT1, MT2	1	-32	1	30.5	20	1.2
	MS1, MS2	0	//	2	78.9	18	-4.1

Results related to reliable sites are presented (LC: Left chest, RC: Right chest, LN: Left neck).

No statistically significant differences between animals showing modulatory effect and no modulatory effect were found when group T and S were compared.

At RC, independently from the group, animals who showed facilitation were largely represented (68% in T and 55% in S).

Concerning the habituation, the only statistically significant difference found was present between MT1 and MT2 at LC (p = 0.03). No other statistical differences were found. Results are reported in the supplementary material (S6 and S7 Tables).

### 4. Other variables

A statistically significant decrease in HR following removal of CS compared to baseline value was recorded in MT1 (p = 0.027) and MT2 (p = 0.024). A statistically significant decrease in body temperature was recorded following the removal of CS compared to baseline in TT (p = 0.024). No other statistically significant differences were found. Results are reported in the supplementary materials (S10 Table).

Feasibility scores results (median and IQR) are reported in supplementary material. No animals had a score of 3 (intolerant to any manipulation). No statistically significant differences were found between and within treatments. Results are reported in the supplementary materials (S11 Table).

Duration of the experimental sessions was: 64 ± 20 minutes (MT1), 54 ± 9 minutes (MT2), 61 ± 10 minutes (MS1), 54 ± 9 minutes (MS2), 77 ± 11 minutes (TT) and 79 ± 14 minutes (TS). No significant differences were found within mechanical (p = 0.360) and thermal (p = 0.921) sessions.

The time needed to record mechanical thresholds after tourniquet removal was 18 ± 7 minutes in MT1, 15 ± 3 minutes in MT2, 19 ± 4 minutes in MS1 and 16 ± 4 MS2. No statistically significant differences were found among sessions (p = 0.118).

The time needed to record thermal thresholds after tourniquet removal was 25 ± 9 minutes for TT and 26 ± 6 minutes for TS. No statistically significant differences were found between sessions (p = 0.974).

## Discussion

The main finding of this trial is the identification mechanical and thermal thresholds that could serve as benchmark for future investigation in this species. Furthermore, among the tested sites, which might be potentially relevant for animals with visceral cardiac pain, the right chest, left chest and left neck were identified as preferential points due to their good to moderate reliability for the sole mechanical thresholds. Additionally, modulation after the conditioning stimulus was seen at the right chest in both groups T and S, with facilitation being predominantly observed.

### 1. Quantitative sensory testing (QST) thresholds

The minipigs enrolled in this study showed high mechanical thresholds; indeed, recorded values were often close or higher than 80 N. The cut-off of stimulation used in this study was chosen based on previous experiences collected by the authors in calves up to 4 months [5], and for the difficulty of maintaining a linear increase in delivering force beyond 80 N. However, considering the present results, it might be opportune in further studies to enlarge this cut-off if the same algometer will be used. Different factors can influence mechanical thresholds. First, increase in age, and consequent increase in body weight, has been shown to correlate with higher mechanical thresholds in pigs [11,13], rats [42,43] and humans [44,45]. Our results suggest that in minipigs age could have a stronger impact compared to body weight, but further studies should be conducted to clarify this issue. Second, the size of the algometer tip has been shown to play a role. In general, smaller algometer tips provoke higher pressure compared to larger ones, and lower force is needed to obtain a response [46]. In piglets, Fosse et al. 2010 found higher thresholds when tips of 0.5–1 cm^2^ were used compared to those recorded with a tip of 0.2 cm^2^ [47]. However, bigger tips were also reported to produce more consistent results [47]. Third, irregularities in the rate of force increase have been indicated as one of other potential factors which cause variability in mechanical thresholds recorded in piglets [13] and in horses [48]. New devices available on the market for detecting mechanical thresholds are equipped with a visual signal which gives real-time feedback if the force exerted by the operator is too high [49]. On the contrary, the manual handheld algometer leaves the responsibility of exerting a constant force to the operator. In our study, all the measurements were performed by one single, previously trained operator, to reduce variability. Fourth, our study was conducted at the farm where the minipigs were hosted with the aim of reducing number of transports and stress. Since most of the experimental sessions were conducted between December and March, the environmental temperature in the experimental room was low (5 ± 5.8°C). Nevertheless, all tests were conducted under infrared heat lamp to mitigate the drop of body temperature and enhance animal comfort. Low environmental temperature (below 8°C) has been shown to play an important role in increasing mechanical thresholds in sheep, presumably due to ischaemia of small nerve fibres and vasoconstriction [50].

The thermal thresholds seldomly reached the cut-off. As for mechanical thresholds, several factors can affect their values. First, environmental temperature seems to have an effect, but controversial results have been reported [51,52]. In our study, we might expect that the low environmental temperature led to vasoconstriction, to a consequent reduction in skin temperature and, therefore, to higher pain tolerance. Unfortunately, we did not measure the skin temperature of our animals before starting stimulation and drawing conclusions would be speculative. Second, the rate of heating increase of the probe could lead to different nociceptor activation. A slow heating rate has been associated with C fibers activation in rats [53]. Therefore, a rate of 0.65 ± 0.13°C/second has been applied in our trial. Third, the presence of subcutaneous fat layer and skin thickness, could lead to high tolerance to the heat stimulus, as skin temperature at which the animals react do not reflect actual temperature perceived at the level of the nociceptors [52]. Forth, as for the mechanical thresholds, increase in age seems to be directly correlated with increase in thresholds [12].

Further studies should be performed in minipigs to highlight potential differences in mechanical and thermal thresholds with the use of different devices and environmental conditions.

### 2. Reliability

For testing ICC between different sessions, test-retest studies are needed, aiming at obtaining repeated measurements using a standard protocol, on the same individuals, under similar circumstances [54]. Analysis of thresholds reliability using ICC has been previously reported in both human and veterinary patients [17,55–63]. A recent study evaluating mechanical pain thresholds measured at the level of the dorsal metacarpal bone of both forelimbs in adolescent female pigs found a moderate ICC (0.45) between sessions [56], which is in line with the reported ICC at LHL in the present study. However, when comparing results in veterinary species with humans, it becomes clear that the reliability of thresholds in our field is lower than that observed with humans. There is an inherent challenge in accurately assessing pain-related responses in animals and distinguishing between pain-related reaction and reactions resulting from discomfort, stress or fear is not always straightforward. To minimise any potential variations, we, marked the exact sites to be stimulated, and assigned only one operator to record thresholds [64]. Moreover, as training has been shown positively influence animals’ response to painful stimulus and improve reliability of the results [57] we tried to habituate the minipigs to the experimental conditions in advance. Despite these precautions, a moderate to good reliability of mechanical thresholds was only found at LC (ICC = 0.59), RC (ICC = 0.83) and LN (ICC = 0.51). This suggest the abovementioned sites to be the most appropriate for future studies assessing mechanical QST in minipigs with potential cardiac visceral pain.

To the best of the authors’ knowledge, the reliability of thermal thresholds in veterinary species has never been investigated so far. However, test-retest reliability of pain elicited by contact heat pain ranged from poor to moderate in humans [65] and with large inter-individual variability [66]. In light of these evidence and of our not encouraging results, one might question their validity and usefulness in animal species.

A considerable number of body sites have been tested, and in particular the ones affected by referred pain in humans suffering of myocardial infarction (except for the hindlimb) [67]. This choice was indeed made to acquire knowledge on QST and CPM in healthy minipigs, as a first step for the assessment of pain in minipigs undergoing experimentally induced myocardial infarction (data not reported in this manuscript).

### 3. Assessment of thresholds after conditioning stimulus

In our trial, minipigs showed prevalence towards a pronociceptive profile (facilitatory effect) following the application of the CS. In the human literature, factors which lead patients to show an antinociceptive phenotype (inhibitory effect) or a pro-nociceptive phenotype (facilitatory effect) include: 1) subjective conditions (e.g. mood, stress, tendency to catastrophising), physiological (e.g. sex, age) and pathological conditions (e.g. chronic or neurodegenerative diseases), and 2) variables related to the methodology (e.g. type and intensity of CS) [21,24,39,62,68–70]. Our results could be explained by the stress-induced hyperalgesia phenomenon [71,72]. The prolonged immobilisation in the sling could have played an important role [73]. However, this drawback seems hard to overcome as the use of a sling specifically made for minipigs should reduce stress related to physical restrain [74], as also acclimatisation and training [73,75]. Another source of stress could have been the isolation from the group [73], as during the tests there was no visual contact with mates.

Results obtained at the RC are suggestive of a modulation linked to the conditioning stimulus. However, this modulatory effect was present both during treatment and sham. This result is not surprising, though. Distraction and stress have been shown to influence pain modulation in humans [72,76]. Tracey and Dunckley (2004) suggested that brain regions implicated in hypervigilance may be linked to brainstem structures responsible for diffuse noxious inhibitory control, potentially leading to dysregulation of the system [77]. In our opinion, distraction due to the perception of the cuff, along with stress, could have had triggered an effect similar to that potentially induced by a painful CS.

Many animals showed no modulatory effect. We hypothesized that the time needed to perform the test following the removal of the conditioning stimulus could have played a role. Indeed, the duration of the CPM effect following application of CS is not clear and it might depend on the type of CS used [61]. Contrasting results about the length of CPM effect induced by the tourniquet have been found in humans. While Fujii et al. (2012) described a CPM effect which lasted only 5 minutes after removal of the tourniquet [78], Tuveson et al. (2006) found it to last for 30 minutes [79]. We took 15 to 19 minutes to complete the mechanical thresholds assessment following CS removal, and around 26 minutes for thermal thresholds assessment. This was due to the numerous sites tested. As the different body sites were tested in a random order, we cannot assess the effect of time at each location, but the randomization itself should have limited the confounding effect of the time. Reducing the time needed to assess CPM and focusing on the reliable sites found in the present study could highlight potential differences. However, absence of modulatory effect, independently from the time needed to assess response following the application of a CS, has been described in both animals and humans [5,25].

Modifications in absolute change of mechanical thresholds between the first and the second tourniquet treatment were absent, except at LC. Habituation has been defined as a decrease in frequency or magnitude of a response due to repeated stimuli despite constant stimulation intensity [80–82]. We consider development of habituation unlikely in this study as removing an outlier from the dataset, not statistically differences were found.

Despite the effort made by the expert in the field, a solid paradigm of CPM has not been established yet in human medicine [83]and different conditioning stimuli have been reported in the literature [84]. The same issue is also present in veterinary medicine, exacerbated by the paucity of studies investigating this phenomenon. In our study, an ischemic stimulus (pneumatic tourniquet) was applied as CS, and the intensity/duration of the stimulus were based on reported recommendations [85]. It is clear that to elicit CPM a mild to moderately painful conditioning stimulus must be warranted, but which conditioning stimulus (e.g. cold or hot water bath, ischaemic pain) is preferrable has not been established yet [21,61,84,86]. Contrasting results about the usefulness of an ischaemic conditioning stimulus in veterinary studies have been found; it was considered unreliable in one study in dogs [28], whereas it was successfully applied in another trial conducted in the same species [29] and in a third one in calves [5]. In the present trial we opted for a tourniquet for two reasons: 1) an ischemic stimulus was in our opinion the most trustable surrogate of the pain derived by a myocardial infarction 2) the tourniquet met the requirement of inducing an ischemic conditioning stimulus under controlled conditions, as time and pressure could be precisely regulated and maintained at a stable level. Additionally, it could generate a reversible and short pain.

There is also no gold standard for reporting results of CPM, but the reporting of absolute and percentage change has been recommended [22]. Moreover, the use of the SEM, which has been proposed by Locke et al. 2014 [25] as a method to establish meaningful CPM effects, has been widely accepted and considered statistically robust and potentially useful for the interpretation of results across different studies [61]. Therefore, we followed these methods in this trial.

For this study, no sample size was calculated a priori; the minipigs enrolled were purchased for being involved in another trial and we had no references for a meaningful calculation. Differences in QST thresholds between sexes could be expected in the present study, as the role of gender has been highlighted in several publications involving humans [87]. However, the statistical analysis was conducted on the entire sample without addressing gender related differences due to the limited sample size.

## Conclusions

In the present study, mechanical and thermal thresholds were established in healthy minipigs, offering reference values for future trials in this species. Mechanical thresholds proved to be more reliable and hence more valuable than thermal thresholds. Minipigs tended to exhibit a pro-nociceptive profile, which could be due to stress-induced hyperalgesia. However, this phenomenon, as well as the absence of modulatory effect in several sites and animals, should be confirmed with a stricter paradigm.

## Supporting information

S1 Fig Graphic description of the minipigs ‘pen and its surrounding areas. (DOCX)

S2 Fig Number of animals exhibiting a modulatory effect (in green) or no-modulatory effect (in blue) after conditioning stimulus at treatment mechanical tourniquet 1 and 2 in tested sites which showed poor reliability. LF: Left forearm, RF: Right forearm, RN: Right neck.
(DOCX)

S3 Fig Number of animals exhibiting a modulatory effect (in green) or no-modulatory effect (in blue) after conditioning stimulus at treatment mechanical sham 1 and 2 in tested sites which showed poor reliability. LF: Left forearm, RF: Right forearm, RN: Right neck.
(DOCX)

S4 Fig Number of animals exhibiting a modulatory effect (in green) or no-modulatory effect (in blue) after conditioning stimulus at treatment thermal tourniquet in all the tested sites. LF: Left forearm, RF: Right forearm, LC: Left chest, RC: Right chest, LN: Left neck, RN: Right neck.
(DOCX)

S5 Fig Number of animals exhibiting a modulatory effect (in green) or no-modulatory effect (in blue) after conditioning stimulus at treatment thermal sham in all the tested sites. LF: Left forearm, RF: Right forearm, LC: Left chest, RC: Right chest, LN: Left neck, RN: Right neck.
(DOCX)

S1 Table Raw mechanical thresholds, before and after the application of the conditioning stimulus (CS); females only. Results (in Newton) are presented as median and interquartile range [25^th^; 75^th^]. Mechanical thresholds are reported in all the tested sites (LHL: Left hindlimb, LF: Left forearm, RF: Right forearm, LC: Left chest, RC: Right chest, LN: Left neck, RN: Right neck) both before and after the application of the CS in all the sessions (MT1: Mechanical tourniquet 1, MT2: Mechanical tourniquet 2, MS1: Mechanical sham 1, MS2: Mechanical sham 2).
(DOCX)

S2 Table Raw mechanical thresholds, before and after the application of the conditioning stimulus (CS); males only. Results (in Newton) are presented as median and interquartile range [25^th^; 75^th^]. Mechanical thresholds are reported in all the tested sites (LHL: Left hindlimb, LF: Left forearm, RF: Right forearm, LC: Left chest, RC: Right chest, LN: Left neck, RN: Right neck) both before and after the application of the CS in all the sessions (MT1: Mechanical tourniquet 1, MT2: Mechanical tourniquet 2, MS1: Mechanical sham 1, MS2: Mechanical sham 2). * One missing value (n = 4).
(DOCX)

S3 Table Raw values of thermal thresholds, before and after the application of the conditioning stimulus (CS), females only. Results (degrees Celsius) are presented as median and interquartile range [25^th^; 75^th^]. Thermal thresholds are reported in all the tested sites (LHL: Left hindlimb, LF: Left forearm, RF: Right forearm, LC: Left chest, RC: Right chest, LN: Left neck, RN: Right neck) both before and after the application of the CS in all the sessions (TT: Thermal tourniquet; TS: Thermal sham). * One missing value (n = 5).
(DOCX)

S4 Table Raw values of thermal thresholds, before and after the application of the conditioning stimulus (CS), males only. Results (degrees Celsius) are presented as median and interquartile range [25^th^; 75^th^]. Thermal thresholds are reported in all the tested sites (LHL: Left hindlimb, LF: Left forearm, RF: Right forearm, LC: Left chest, RC: Right chest, LN: Left neck, RN: Right neck) both before and after the application of the CS in all the sessions (TT: Thermal tourniquet; TS: Thermal sham).
(DOCX)

S5 Table Number of responders (R) and non-responders (NR) to Von Frey filaments per each tested site and treatment (MT1: Mechanical tourniquet 1; MT2: Mechanical tourniquet 2; MS1: Mechanical sham 1; MS2: Mechanical sham 2; TT: Thermal tourniquet; TS: Thermal sham). The filament to which the animals responded is reported. LHL: Left hindlimb, LF: Left forearm, RF: Right forearm, LC: Left chest, RC: Right chest, LN: Left neck, RN: Right neck. CS = conditioning stimulus.
(DOCX)

S6 Table Absolute and % change (n = 11) calculated for each time point and treatment and relative p value (comparison of absolute changes between MT1 and MS1 and MT2 and MS2). Results are presented as median and interquartile range [25th; 75th]. LHL: Left hindlimb, LF: Left forearm, RF: Right forearm, LC: Left chest, RC: Right chest, LN: Left neck, RN: Right neck.
(DOCX)

S7 Table Absolute and % change (n = 11) calculated for each site and treatment and relative p value (comparison of absolute changes between thermal tourniquet (TT) and thermal sham (TS). Results are presented as median and interquartile range [25th; 75th]. LF: Left forearm, RF: Right forearm, LC: Left chest, RC: Right chest, LN: Left neck, RN: Right neck.
(DOCX)

S8 Table Modulatory effect (2*SEM) calculated for each site and treatment (T: Tourniquet and S: Sham), for both mechanical (MT1 and 2, MS1 and 2) and thermal thresholds. LF: Left forearm, RF: Right forearm, LC: Left chest, RC: Right chest, LN: Left neck, RN: Right neck.
(DOCX)

S9 Table Number of minipigs which had facilitation, inhibition or no effect and relative mean absolute change in sites which showed poor reliability at treatment mechanical tourniquet 1 and 2 (MT1 and MT2) and mechanical sham 1 and 2 (MS1 and MS2). LF: Left forearm, RF: Right forearm, RN: Right neck.
(DOCX)

S10 Table Results of heart rate (HR), respiratory rate (RR) and temperature (T) recorded for each treatment (MT1 = mechanical tourniquet 1, MT2 = mechanical tourniquet 2, MS1 = mechanical sham 1, MS2 = mechanical sham 2, TT = thermal tourniquet, TS = thermal sham) and relative p value. CS = conditioning stimulus. Results are reported as median and interquartile range [25^th^, 75^th^].
(DOCX)

S11 Table Feasibility scores median and interquartile range (IQR, 25th– 75th) for each treatment (mechanical- tourniquet 1, mechanical tourniquet 2, mechanical sham 1, mechanical sham 2, thermal tourniquet and thermal sham) and relative p value. CS = conditioning stimulus.
(DOCX)
