# Supplementary figures and images for: Mechanical and thermal thresholds before and after application of a conditioning stimulus in healthy Göttingen Minipigs
Source: PLoS One. 2024 Aug 29;19(8):e0309604. doi: 10.1371/journal.pone.0309604 (PMC11361583; doi:10.1371/journal.pone.0309604)

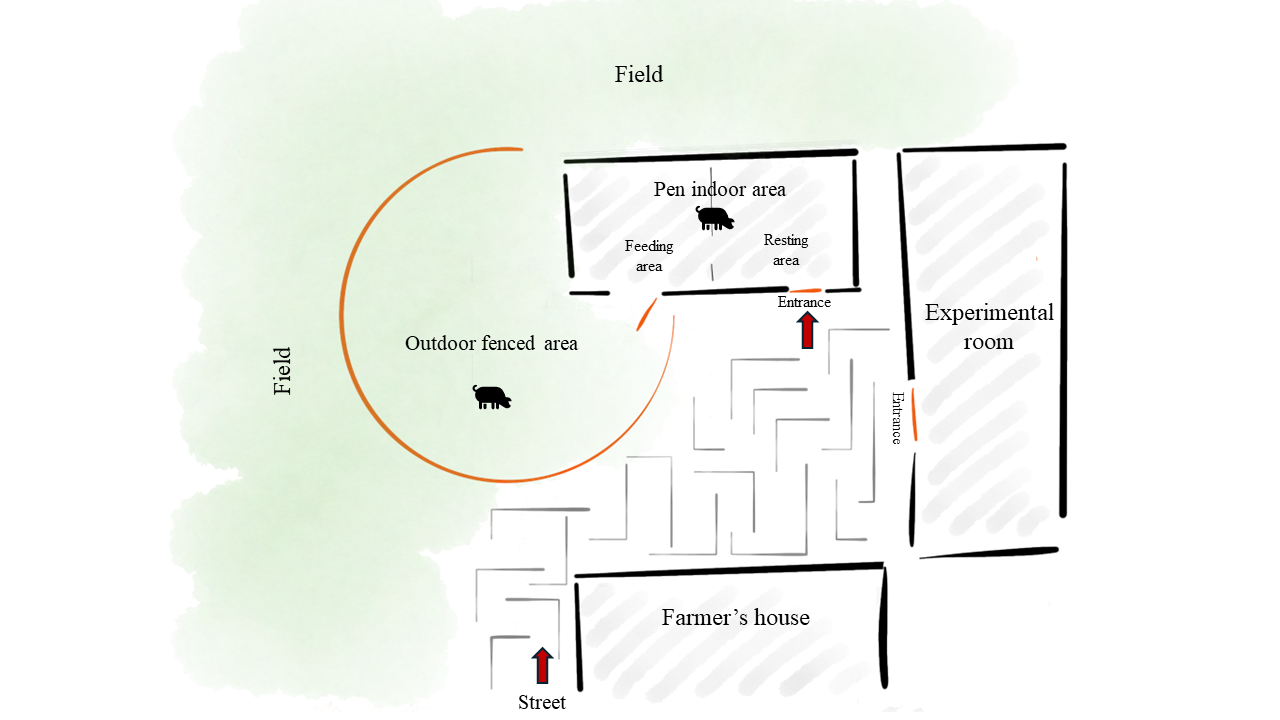

Supplement: S1 Fig — (DOCX) [file pone.0309604.s001.docx]

**
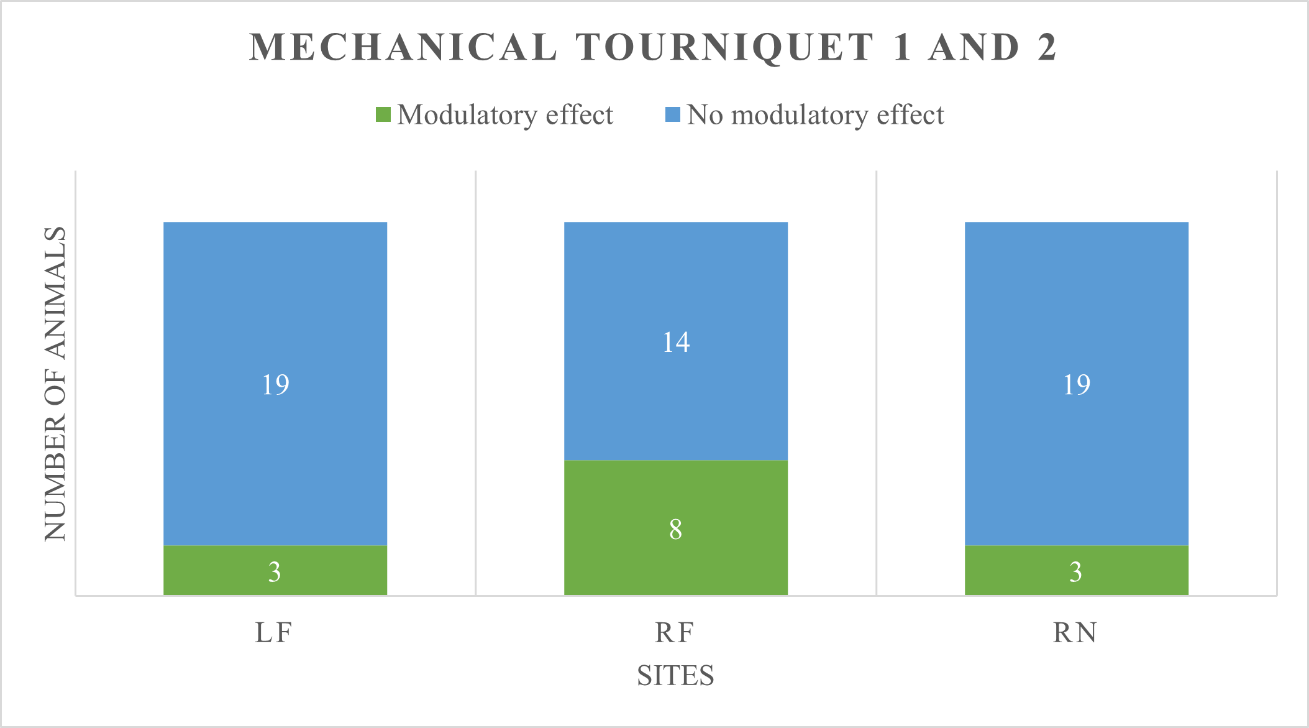
**

Supplement: S2 Fig — LF: Left forearm, RF: Right forearm, RN: Right neck. (DOCX) [file pone.0309604.s002.docx]

**
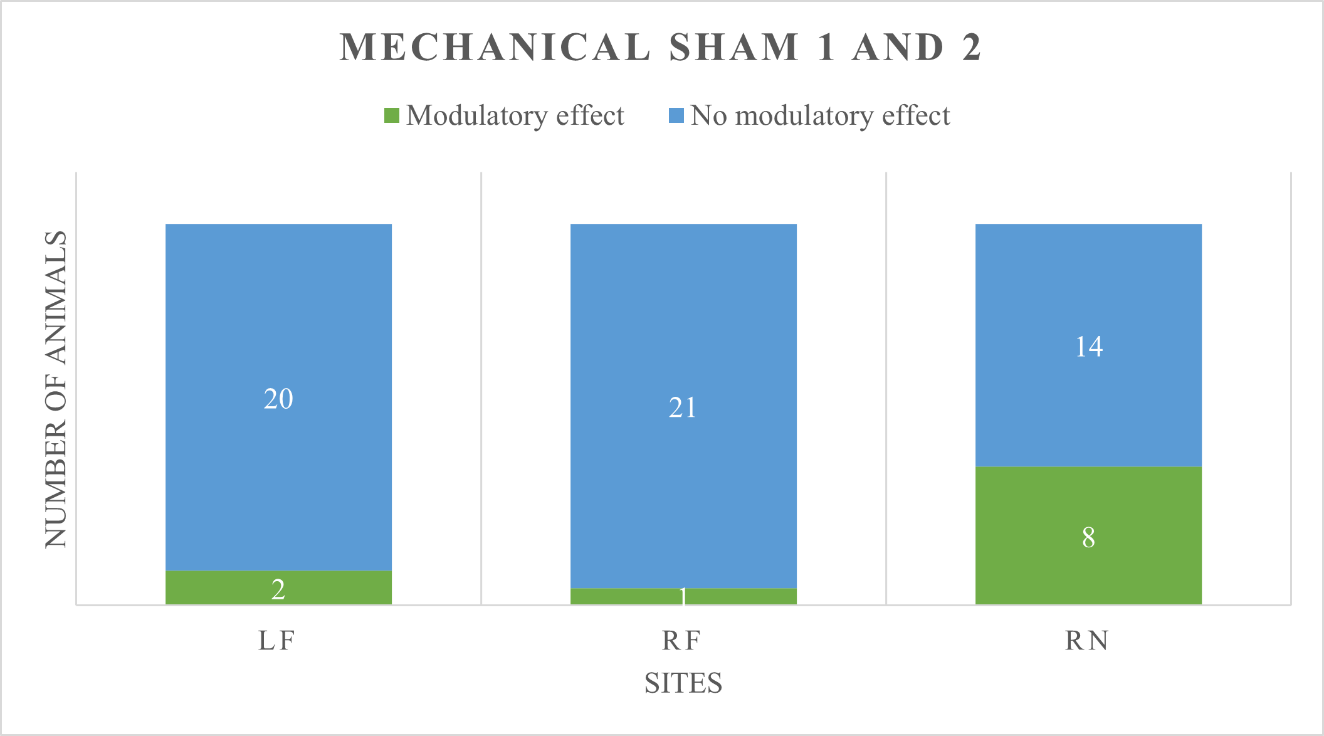
**

Supplement: S3 Fig — LF: Left forearm, RF: Right forearm, RN: Right neck. (DOCX) [file pone.0309604.s003.docx]

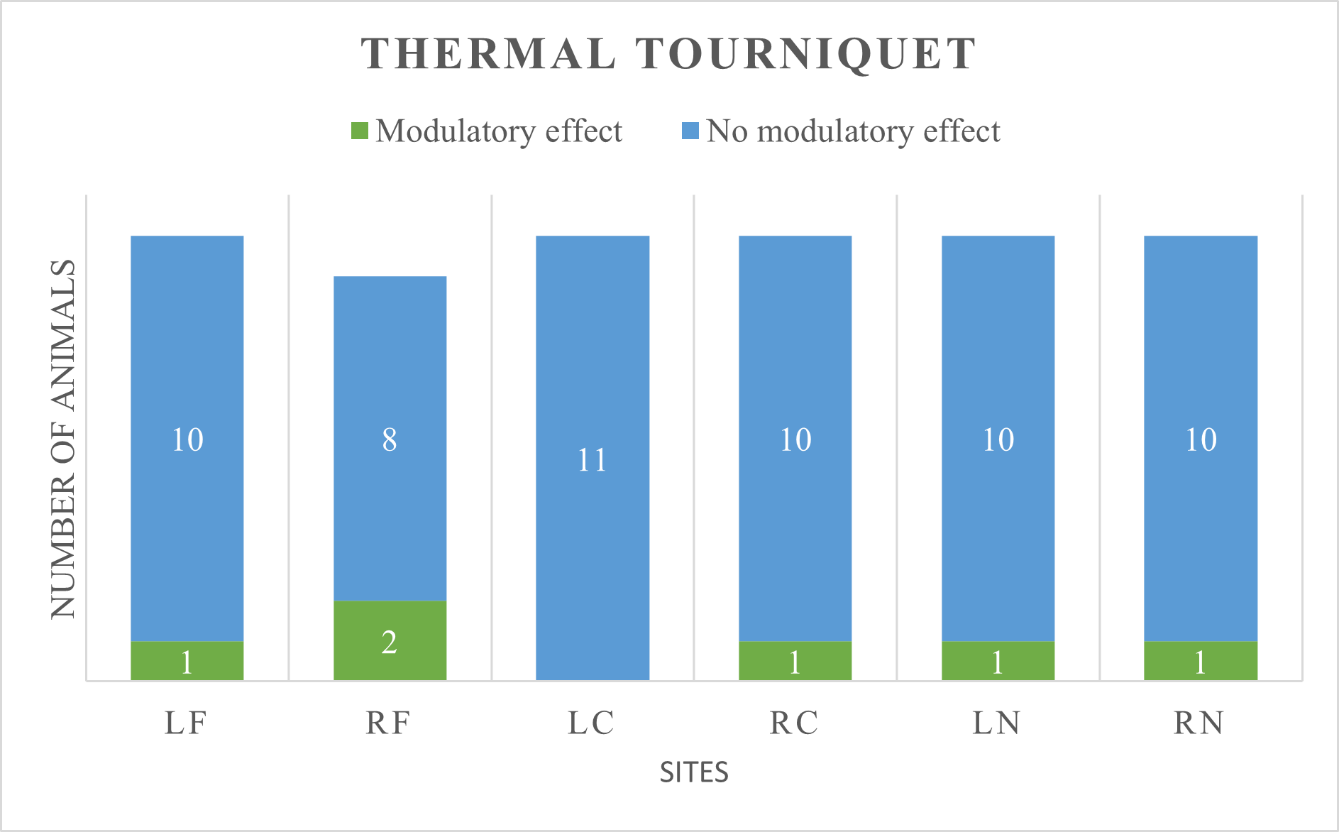

Supplement: S4 Fig — LF: Left forearm, RF: Right forearm, LC: Left chest, RC: Right chest, LN: Left neck, RN: Right neck. (DOCX) [file pone.0309604.s004.docx]

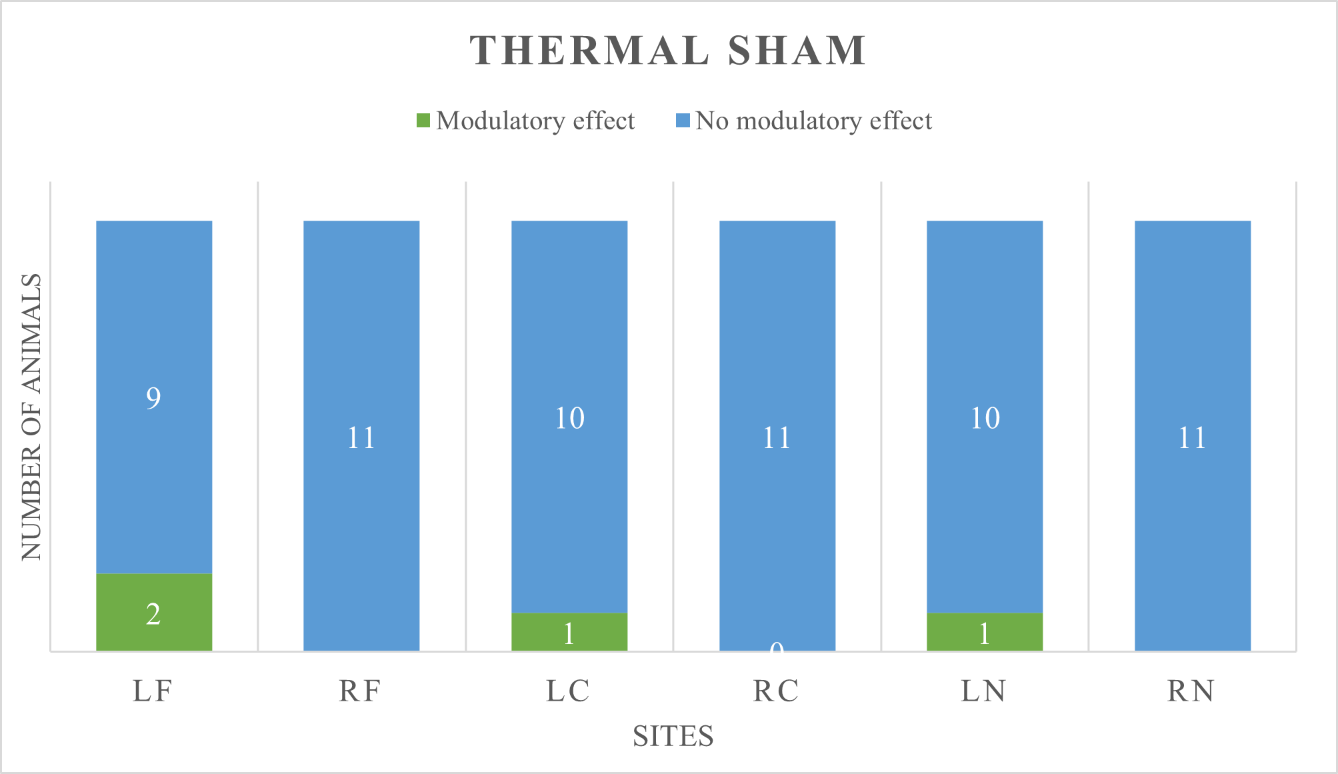

Supplement: S5 Fig — LF: Left forearm, RF: Right forearm, LC: Left chest, RC: Right chest, LN: Left neck, RN: Right neck. (DOCX) [file pone.0309604.s005.docx]
