# Supplementary material for: Mechanical and thermal thresholds before and after application of a conditioning stimulus in healthy Göttingen Minipigs
Source: PLoS One. 2024 Aug 29;19(8):e0309604. doi: 10.1371/journal.pone.0309604 (PMC11361583; doi:10.1371/journal.pone.0309604)
Supplement: S1 Table — Results (in Newton) are presented as median and interquartile range [25th; 75th]. Mechanical thresholds are reported in all the tested sites (LHL: Left hindlimb, LF: Left forearm, RF: Right forearm, LC: Left chest, RC: Right chest, LN: Left neck, RN: Right neck) both before and after the application of the CS in all the sessions (MT1: Mechanical tourniquet 1, MT2: Mechanical tourniquet 2, MS1: Mechanical sham 1, MS2: Mechanical sham 2). (DOCX) [file pone.0309604.s006.docx]

| **Females** | | | | | |
| --- | --- | --- | --- | --- | --- |
| **SITE** | **Time point** | **MT1**  (n=6) | **MT2**  (n=6) | **MS1**  (n=6) | **MS2**  (n=6) |
| **LHL** | Before CS | 81  [72.1; 81] | 81  [55.9; 81] | 81  [78.5; 81] | 72.8  [61.1; 81] |
|  | After  CS | 81  [77.1; 81] | 63.3  [51; 80.6] | 74.5  [65.4; 81] | 74  [53.6; 80.3] |
| **LF** | Before CS | 81  [78.4; 81] | 73.5  [57.9; 81] | 81  [78.8; 81] | 78.8  [50.3; 81] |
|  | After  CS | 81  [77.1; 81] | 81  [49.1; 81] | 81  [79; 81] | 81  [78.5; 81] |
| **RF** | Before CS | 81  [78.1; 81] | 79.5  [73.8; 81] | 81  [74; 81] | 80.8  [73.4; 81] |
|  | After  CS | 78  [69; 81] | 80.8  [51.5; 81] | 81  77,6; 81] | 81  [72.9; 81] |
| **LC** | Before CS | 71.8  [44.9; 80] | 79.5  [67.9; 81] | 81  [59.5; 81] | 81  [71.1; 81] |
|  | After  CS | 76  [71.3; 79.5] | 52.3  [36.5; 77.4] | 67.5  [60; 75.9] | 72.8  [53.9; 80.6] |
| **RC** | Before CS | 81  [75; 81] | 81  [72.3; 81] | 79.3  [68.5; 81] | 81  [66.1; 81] |
|  | After  CS | 58.5  [36.4; 78.4] | 67.8  [57.3; 71.3] | 78.3  [64.5; 79.9] | 75.5  [65.6; 81] |
| **LN** | Before CS | 68.8  [59.3; 81] | 78.8  [65.1; 81] | 81  [80.4; 81] | 81  [58.5; 81] |
|  | After  CS | 75  [62.5; 79.6] | 69.3  [56.3; 81] | 77  [71.9; 81] | 73.8  [57.3; 81] |
| **RN** | Before CS | 73.8  [66.3; 81] | 72.5  [33.3; 76.8] | 80.8  [65.3; 81] | 76  [63.1; 81] |
|  | After  CS | 72.3  [54.1; 78.8] | 66.3  [41.6; 77.3] | 72.8  [62.3; 81] | 74.3  [54.5; 81] |
