# Supplementary material for: Mechanical and thermal thresholds before and after application of a conditioning stimulus in healthy Göttingen Minipigs
Source: PLoS One. 2024 Aug 29;19(8):e0309604. doi: 10.1371/journal.pone.0309604 (PMC11361583; doi:10.1371/journal.pone.0309604)
Supplement: S2 Table — Results (in Newton) are presented as median and interquartile range [25th; 75th]. Mechanical thresholds are reported in all the tested sites (LHL: Left hindlimb, LF: Left forearm, RF: Right forearm, LC: Left chest, RC: Right chest, LN: Left neck, RN: Right neck) both before and after the application of the CS in all the sessions (MT1: Mechanical tourniquet 1, MT2: Mechanical tourniquet 2, MS1: Mechanical sham 1, MS2: Mechanical sham 2). * One missing value (n = 4). (DOCX) [file pone.0309604.s007.docx]

| **Males** | | | | | |
| --- | --- | --- | --- | --- | --- |
| **SITE** | **Time point** | **MT1**  (n=5) | **MT2**  (n=5) | **MS1**  (n=5) | **MS2**  (n=5) |
| **LHL** | Before CS* | 81  [80.6; 81] | 78  [69.5; 80.5] | 81  [79.8; 81] | 81  [59.3; 81] |
|  | After  CS | 71.5  [58.3; 80.5] | 66.5  [48.8; 68] | 57.5  [53.3; 63] | 71  [48.3; 71.8] |
| **LF** | Before CS | 81  [89.3; 81] | 79  [76.8; 81] | 81  [70.8; 81] | 81  [63.5; 81] |
|  | After  CS | 71.5  [58.3; 80.5] | 80  [67.8; 80.8] | 78  [70.3; 80.5] | 80  [73.3; 81] |
| **RF** | Before CS | 81  [81; 81] | 75.5  [66; 80] | 80.5  [67.3; 81] | 81  [66.3; 81] |
|  | After  CS | 69.5  [60; 73.5] | 67.5  [59.5; 75.8] | 74  [73.5; 78] | 71  [50.5; 79] |
| **LC** | Before CS | 81  [69.5; 81] | 80  [52.8; 81] | 80.5  [74.3; 81] | 74.5  [61; 80.8] |
|  | After  CS | 81  [73; 81] | 66  [61.8; 81] | 77.5  [46.8; 78.8] | 59  [47.8; 76.5] |
| **RC** | Before CS | 80.5  [72.5; 81] | 81  [70.5; 81] | 81  [76.8; 81] | 81  [78.8; 81] |
|  | After  CS | 78  [60.3; 81] | 60  [47.3; 72] | 63  [43.1; 77.8] | 67  [51; 76.3] |
| **LN** | Before CS | 80.5  [69.3; 81] | 71  [50.8; 81] | 81  [79.3; 81] | 81  [69.3; 81] |
|  | After  CS | 81  [77.5; 81] | 81  [62; 81] | 78.5  [62.3; 80.3] | 78.5  [75; 81] |
| **RN** | Before CS | 75.5  [55.8; 79.8] | 66  [53.8; 76.8] | 65.5  [58.8; 78.8] | 74.5  [55.8; 79] |
|  | After  CS | 80.5  [69; 80.8] | 63.5  [56.3; 74.3] | 79  [54.5; 81] | 80  [68.3; 81] |
