# Supplementary material for: Mechanical and thermal thresholds before and after application of a conditioning stimulus in healthy Göttingen Minipigs
Source: PLoS One. 2024 Aug 29;19(8):e0309604. doi: 10.1371/journal.pone.0309604 (PMC11361583; doi:10.1371/journal.pone.0309604)
Supplement: S3 Table — Results (degrees Celsius) are presented as median and interquartile range [25th; 75th]. Thermal thresholds are reported in all the tested sites (LHL: Left hindlimb, LF: Left forearm, RF: Right forearm, LC: Left chest, RC: Right chest, LN: Left neck, RN: Right neck) both before and after the application of the CS in all the sessions (TT: Thermal tourniquet; TS: Thermal sham). * One missing value (n = 5). (DOCX) [file pone.0309604.s008.docx]

| **Females** | | | |  |
| --- | --- | --- | --- | --- |
| **SITE** | **Time point** | **TT (n=6)** | **TS (n=6)** |  |
| **LHL** | Before CS | 44.8 [43; 47.9] | 48.6 [45.3; 56] |  |
|  | After CS | 45.7 [44.6-47.3] | 50.7 [45.4; 55.1] |  |
| **LF** | Before CS | 50.4 [44.9; 52.3] | 48 [44.5; 51.2] |  |
|  | After CS | 48 [44; 52.4] | 50.8 [44.8; 55.3] |  |
| **RF** | Before CS | 48.8 [45.1; 56] | 47.3 [44.7; 53] |  |
|  | After CS* | 45.5 [42.5; 56] | 55.5 [47.5; 56] |  |
| **LC** | Before CS | 46 [41.8; 55.3] | 44.8 [42.9; 50.2] |  |
|  | After CS | 44 [42.8; 51.4] | 43.5 [40.4; 46.4] |  |
| **RC** | Before CS | 46.7 [43.3; 52] | 45.6 [43.5; 48.5] |  |
|  | After CS | 45.5 [43.2; 49.6] | 45.3 [44; 48.4] |  |
| **LN** | Before CS | 44.8 [44.1; 51.5] | 45.5 [44.2; 47.5] |  |
|  | After CS | 45 [42.4; 48.1] | 47.3 [46.1; 48.6] |  |
| **RN** | Before CS | 47.9 [43.9, 52] | 47.2 [44.7; 55] |  |
|  | After CS | 53.3 [48.1; 56] | 48 [45.4; 50.6] |  |
