# Supplementary material for: Mechanical and thermal thresholds before and after application of a conditioning stimulus in healthy Göttingen Minipigs
Source: PLoS One. 2024 Aug 29;19(8):e0309604. doi: 10.1371/journal.pone.0309604 (PMC11361583; doi:10.1371/journal.pone.0309604)
Supplement: S4 Table — Results (degrees Celsius) are presented as median and interquartile range [25th; 75th]. Thermal thresholds are reported in all the tested sites (LHL: Left hindlimb, LF: Left forearm, RF: Right forearm, LC: Left chest, RC: Right chest, LN: Left neck, RN: Right neck) both before and after the application of the CS in all the sessions (TT: Thermal tourniquet; TS: Thermal sham). (DOCX) [file pone.0309604.s009.docx]

| **Males** | | | |  |
| --- | --- | --- | --- | --- |
| **SITE** | **Time point** | **TT (n=6)** | **TS (n=6)** |  |
| **LHL** | Before CS | 45.4 [43.8; 46.5] | 48.5 [41.9; 50.6] |  |
|  | After CS | 44.7 [43.6; 50.9] | 44.6 [43.5; 48.3] |  |
| **LF** | Before CS | 48.7 [46.4; 53] | 49 [46.6; 53.4] |  |
|  | After CS | 46.6 [44.3; 51.3] | 44.3 [44; 47.8 |  |
| **RF** | Before CS | 53.8 [43.2; 55.5] | 55.1 [47.8; 56] |  |
|  | After CS | 54.4 [42.2; 55.5] | 49.7 [44.8; 53.6] |  |
| **LC** | Before CS | 45.3 [41.7; 47.2] | 45.3 [43; 45.1] |  |
|  | After CS | 44.3 [42.4; 45.3] | 43 [42; 44.3] |  |
| **RC** | Before CS | 45.2 [43; 45.7] | 43.1 [42.6; 45.8] |  |
|  | After CS | 44.3 [42.2; 47.8] | 43.4 [40.8; 43.6] |  |
| **LN** | Before CS | 44.2 [43.5; 49.3] | 47.4 [44.4; 48.5] |  |
|  | After CS | 47.7 [43; 51.3] | 44.6 [42.3; 47.5] |  |
| **RN** | Before CS | 47.4 [45.5; 52.8] | 51.5 [44.5; 55] |  |
|  | After CS | 45.5 [43.3; 48.6] | 51.6 [41.7; 55] |  |
