# Supplementary material for: Mechanical and thermal thresholds before and after application of a conditioning stimulus in healthy Göttingen Minipigs
Source: PLoS One. 2024 Aug 29;19(8):e0309604. doi: 10.1371/journal.pone.0309604 (PMC11361583; doi:10.1371/journal.pone.0309604)
Supplement: S5 Table — The filament to which the animals responded is reported. LHL: Left hindlimb, LF: Left forearm, RF: Right forearm, LC: Left chest, RC: Right chest, LN: Left neck, RN: Right neck. CS = conditioning stimulus. (DOCX) [file pone.0309604.s010.docx]

**Supplementary table 5. *Number of responders (R) and non-responders (NR) to Von Frey filaments per each tested site and treatment*** (MT1: mechanical tourniquet 1; MT2: mechanical tourniquet 2; MS1: mechanical sham 1; MS2: mechanical sham 2; TT: thermal tourniquet; TS: thermal sham). The filament to which the animals responded is reported. LHL: left hindlimb, LF: left forearm, RF: right forearm, LC: left chest, RC: right chest, LN: left neck, RN: right neck. CS= conditioning stimulus.

|  | | **MT1 and MT2** | | **MS1 and MS2** | | **TT** | | **TS** | |
| --- | --- | --- | --- | --- | --- | --- | --- | --- | --- |
| **Site** | **Time point** | **R** | **NR** | **R** | **NR** | **R** | **NR** | **R** | **NR** |
| **LHL** | *Before*  *CS* | 0 | 22 | 1  (300 g/cm^2^) | 21 | 0 | 11 | 0 | 11 |
|  | *After*  *CS* | 0 | 22 | 0 | 22 | 0 | 11 | 0 | 11 |
| **LF** | *Before*  *CS* | 0 | 22 | 0 | 22 | 0 | 11 | 0 | 11 |
|  | *After*  *CS* | 0 | 22 | 0 | 22 | 0 | 11 | 0 | 11 |
| **RF** | *Before*  *CS* | 0 | 22 | 0 | 22 | 0 | 11 | 0 | 11 |
|  | *After*  *CS* | 1  (60 g/cm^2^) | 21 | 0 | 22 | 0 | 11 | 0 | 11 |
| **LC** | *Before*  *CS* | 0 | 22 | 0 | 22 | 0 | 11 | 0 | 11 |
|  | *After*  *CS* | 1  (180 g/cm^2^) | 21 | 0 | 22 | 0 | 11 | 0 | 11 |
| **RC** | *Before*  *CS* | 1  (300 g/cm^2^) | 21 | 0 | 22 | 0 | 11 | 0 | 11 |
|  | *After*  *CS* | 0 | 22 | 0 | 22 | 0 | 11 | 1  (180 g/cm^2^) | 10 |
| **LN** | *Before*  *CS* | 0 | 22 | 0 | 22 | 0 | 11 | 1  (26 g/cm^2^) | 10 |
|  | *After*  *CS* | 0 | 22 | 0 | 22 | 0 | 11 | 0 | 11 |
| **RN** | *Before*  *CS* | 0 | 22 | 0 | 22 | 0 | 11 | 0 | 11 |
|  | *After*  *CS* | 0 | 22 | 0 | 22 | 0 | 11 | 0 | 11 |
