# Supplementary material for: Mechanical and thermal thresholds before and after application of a conditioning stimulus in healthy Göttingen Minipigs
Source: PLoS One. 2024 Aug 29;19(8):e0309604. doi: 10.1371/journal.pone.0309604 (PMC11361583; doi:10.1371/journal.pone.0309604)
Supplement: S6 Table — Results are presented as median and interquartile range [25th; 75th]. LHL: Left hindlimb, LF: Left forearm, RF: Right forearm, LC: Left chest, RC: Right chest, LN: Left neck, RN: Right neck. (DOCX) [file pone.0309604.s011.docx]

| **SITES** | **Treatment** | **ABSOLUTE CHANGE** | **P value**  **(MT1 vs MT2)** | **P value**  **(MS1 vs MS2)** | **% CHANGE** |
| --- | --- | --- | --- | --- | --- |
| LF | MT1 | 0 [-5; 0] | 0.739 | 0.262 | 0 [-7,09; 0] |
|  | MS1 | 0 [-1; 1] |  |  | 0 [-1.2; 1.4] |
|  | MT2 | -0.5 [-11.5; 5.5] |  |  | -0.6 [-14.6; 7.3] |
|  | MS2 | 0 [0; 19.5] |  |  | 0 [0; 31.7] |
| RF | MT1 | -6.5 [-11.5; -1] | 0.339 | 0.388 | -9.4 [-14.2; -1.2] |
|  | MS1 | 0 [-2; 2.5] |  |  | 0 [-2.7; 3.2] |
|  | MT2 | -3.5 [-12; 0.5] |  |  | -4.7 [-15.2; 0.62] |
|  | MS2 | -1 [-10; 0] |  |  | -1.9 [-12.4; 0] |
| LC | MT1 | 1.5 [0; 12.5] | **0.03** | 0.869 | 1.9 [0; 18.6] |
|  | MS1 | -3.5 [-15.5; -1] |  |  | -4.3 [-19.1; -1.2] |
|  | MT2 | -2.5 [-21.5; 1] |  |  | -6.7 [-25.54; 1.25] |
|  | MS2 | -3.5 [-21.5; 0.5] |  |  | -4.3 [-26.5; 0.62] |
| RC | MT1 | -3 [-26; 0] | 0.264 | 0.921 | -3.7 [-35.8; 0] |
|  | MS1 | -4 [-18; 3] |  |  | -4.9 [-22.2; 4.1] |
|  | MT2 | -13.5 [-21; -10] |  |  | -18.5 [-29.4; -12.4] |
|  | MS2 | -9 [-22.5; 0] |  |  | -11.1 [-27.8; 0] |
| LN | MT1 | 0.5 [-1.5; 10] | 0.817 | 0.221 | 0.6 [-1.9; 14.3] |
|  | MS1 | -3 [-12.5; 0] |  |  | -3.7 [-15.4; 0] |
|  | MT2 | 0 [-4.5; 10] |  |  | 0 [-5.6; 18.8] |
|  | MS2 | 0 [-5; 1] |  |  | 0 [-6.2; 1.3] |
| RN | MT1 | 0 [-10; 5] | 0.554 | 0.818 | 0 [-12.4; 6.62] |
|  | MS1 | 0 [-16.5; 8.5] |  |  | 0 [-20.4; 11.7] |
|  | MT2 | 0.5 [-5.5; 7.5] |  |  | 0.7 [-8.3; 16.7] |
|  | MS2 | 2 [-15; 10] |  |  | 2.6 [-21.6; 14.1] |
