# Supplementary material for: Mechanical and thermal thresholds before and after application of a conditioning stimulus in healthy Göttingen Minipigs
Source: PLoS One. 2024 Aug 29;19(8):e0309604. doi: 10.1371/journal.pone.0309604 (PMC11361583; doi:10.1371/journal.pone.0309604)
Supplement: S7 Table — Results are presented as median and interquartile range [25th; 75th]. LF: Left forearm, RF: Right forearm, LC: Left chest, RC: Right chest, LN: Left neck, RN: Right neck. (DOCX) [file pone.0309604.s012.docx]

| **SITES** | **Treatment** | **ABSOLUTE CHANGE** | **P value**  **(TT vs TS)** | **% CHANGE** |
| --- | --- | --- | --- | --- |
| **LF** | TT | -0.8 [-7; 2] | 0.818 | - 1.68 [-13.2; 4.4] |
|  | TS | -1.2 [-5; 2.7] |  | - 2.64 [-10.2; 5.2] |
| **RF** | TT | 0.5 [-5.2; 3.0] | 0.622 | 1.09 [-9.2; 5.8] |
|  | TS | 0.6 [-5.7; 7.8] |  | 1.15 [-10.9; 16.2] |
| **LC** | TT | -1.2 [-4.7; 2.4] | 0.599 | -2.64 [-9.10; 5.73] |
|  | TS | -0.5 [-1.5; 0.4] |  | -1.10 [-3.3; 0.92] |
| **RC** | TT | 0 [-1.6; 2.3] | 0.742 | 0 [-3.5; 4.8] |
|  | TS | -0.2 [-2.1; 0.7] |  | -0.43 [-4.27; 1.64] |
| **LN** | TT | -0.3 [-2.4; 0.7] | 0.869 | -0.67 [-5.43; 1.61] |
|  | TS | -1 [-2.7; 3.1] |  | -2.19 [-5.5; 6.83] |
| **RN** | TT | 0 [-1.9; 1] | 0.947 | 0 [-4.31; 2.18] |
|  | TS | 0 [-0.2; 0.1] |  | 0 [-0.45; 0.21] |
