# Supplementary material for: Mechanical and thermal thresholds before and after application of a conditioning stimulus in healthy Göttingen Minipigs
Source: PLoS One. 2024 Aug 29;19(8):e0309604. doi: 10.1371/journal.pone.0309604 (PMC11361583; doi:10.1371/journal.pone.0309604)
Supplement: S8 Table — LF: Left forearm, RF: Right forearm, LC: Left chest, RC: Right chest, LN: Left neck, RN: Right neck. (DOCX) [file pone.0309604.s013.docx]

| **Site** | **Mechanical**  **(1 and 2)** | **Modulatory effect = 2*SEM** | **Thermal** | **Modulatory effect= 2*SEM** |
| --- | --- | --- | --- | --- |
| *LF* | T | **± 20.9** | T | **± 9.3** |
|  | S | **± 19.6** | S | **± 7.9** |
| *RF* | T | **± 9.6** | T | **± 10.3** |
|  | S | **± 19.8** | S | **± 32.8** |
| ***LC*** | T | **± 19.4** | T | **± 29.2** |
|  | S | **± 12** | S | **± 7.5** |
| ***RC*** | T | **± 6.9** | T | **± 7.5** |
|  | S | **± 5.8** | S | **± 4.8** |
| ***LN*** | T | **± 17.3** | T | **± 6.7** |
|  | S | **± 24.3** | S | **± 3.9** |
| *RN* | T | **± 22.5** | T | **± 12** |
|  | S | **± 20.2** | S | **± 12.5** |
