# Supplementary material for: Mechanical and thermal thresholds before and after application of a conditioning stimulus in healthy Göttingen Minipigs
Source: PLoS One. 2024 Aug 29;19(8):e0309604. doi: 10.1371/journal.pone.0309604 (PMC11361583; doi:10.1371/journal.pone.0309604)
Supplement: S9 Table — LF: Left forearm, RF: Right forearm, RN: Right neck. (DOCX) [file pone.0309604.s014.docx]

| **Site** | **Treatment** | **FACILITATION** | | **INHIBITION** | | **NO EFFECT** | |
| --- | --- | --- | --- | --- | --- | --- | --- |
|  |  | **Number** | **Mean absolute change** | **Number** | **Mean absolute change** | **Number** | **Mean absolute change** |
| **LF** | MT1, MT2 | 2 | -27 | 1 | 21 | 19 | -0.4 |
|  | MS1, MS2 | 0 | // | 2 | 29.8 | 20 | 0.9 |
| **RF** | MT1, MT2 | 8 | -16.3 | 0 | // | 14 | -1.9 |
|  | MS1, MS2 | 1 | -31 | 0 | // | 21 | -0.5 |
| **RN** | MT1, MT2 | 1 | -36.5 | 2 | 31.25 | 19 | -1 |
|  | MS1, MS2 | 4 | -24.8 | 4 | 30.4 | 14 | 0.1 |
