# Supplementary material for: Mechanical and thermal thresholds before and after application of a conditioning stimulus in healthy Göttingen Minipigs
Source: PLoS One. 2024 Aug 29;19(8):e0309604. doi: 10.1371/journal.pone.0309604 (PMC11361583; doi:10.1371/journal.pone.0309604)
Supplement: S10 Table — CS = conditioning stimulus. Results are reported as median and interquartile range [25th, 75th]. (DOCX) [file pone.0309604.s015.docx]

| **Treatment** | **Time point** | **HR**  (beats per minute) | **RR**  (breath per minute) | **Temperature**  (°Celsius) |
| --- | --- | --- | --- | --- |
| **MT1** | Before CS | 124 [112; 140] | 28 [24; 36] | 38.1 [37.8; 38.4] |
|  | After CS | 106 [100; 120] | 24 [20; 32] | 38 [37.3; 38.4] |
| **p value** | | **0.027** | 0.084 | 0.175 |
| **MT2** | Before CS | 128 [100; 144] | 28 [24; 32] | 38.1 [37; 38.6] |
|  | After CS | 104 [96; 120] | 28 [24; 30] | 37.9 [37.2; 38.5] |
| **p value** | | **0.024** | 0.164 | 0.557 |
| **MS1** | Before CS | 120 [100; 124] | 28 [20; 36] | 37.9 [37.6; 38.9] |
|  | After CS | 112 [108; 138] | 24 [20; 32] | 38 [37.9; 38.2] |
| **p value** | | 0.426 | 0.374 | 0.625 |
| **MS2** | Before CS | 132 [108; 140] | 28 [24; 28] | 38.2 [37.1; 38.4] |
|  | After CS | 120 [92; 142] | 24 [24; 28] | 37.8 [36.9; 38.5] |
| **p value** | | 0.322 | 0.742 | 0.820 |
| **TT** | Before CS | 104 [100; 116] | 28 [24; 48] | 38.2 [38; 38.5] |
|  | After CS | 96 [90; 112] | 30 [24; 36] | 37.6 [37.5; 37.9] |
| **p value** | | 0.426 | 0.820 | **0.024** |
| **TS** | Before | 120 [108; 128] | 28 [21; 48] | 38 [37.5; 38.7] |
|  | After | 110 [104; 118] | 28 [20; 32] | 38.4 [37.2; 39] |
| **p value** | | 0.123 | 0.105 | 0.638 |
