# Supplementary material for: Mechanical and thermal thresholds before and after application of a conditioning stimulus in healthy Göttingen Minipigs
Source: PLoS One. 2024 Aug 29;19(8):e0309604. doi: 10.1371/journal.pone.0309604 (PMC11361583; doi:10.1371/journal.pone.0309604)
Supplement: S11 Table — CS = conditioning stimulus. (DOCX) [file pone.0309604.s016.docx]

|  | **Tourniquet (T)** | | **Sham (S)** | | **p-value**  **(T versus S)** |
| --- | --- | --- | --- | --- | --- |
|  | **Median** | **IQR** | **Median** | **IQR** |  |
| **MECHANICAL 1** | | | | | |
| Before CS | 1 | [0; 1] | 1 | [0; 1] | 0,826 |
| After CS | 1 | [0; 1] | 1 | [0; 2] | 0,410 |
| **p-value**  **(Before CS versus After CS)** | 1,000 | | 0,563 | |  |
| **MECHANICAL 2** | | | | | |
| Before CS | 1 | [0; 1] | 0 | [0; 0] | 0,613 |
| After CS | 1 | [1; 2] | 1 | [1; 1] | 0,514 |
| **p-value**  **(Before CS versus After CS)** | 0, 125 | | 0,125 | |  |
| **THERMAL** | | | | | |
| Before CS | 0 | [0; 1] | 1 | [0; 1] | 0,882 |
| After CS | 1 | [0; 1] | 1 | [0; 1] | 0,971 |
| **p-value**  **(Before CS versus After CS)** | 0,313 | | 0,250 | |  |
